# A symptomatic cystic axillary mass in a post-partum female

**DOI:** 10.1093/jscr/rjad033

**Published:** 2023-02-14

**Authors:** Nicole Werwie, Jodi Oostra, Kelly Hanley

**Affiliations:** General Surgery, Ohio Health Riverside Methodist Hospital, Columbus, OH, USA; Breast Surgery, Ohio Health Riverside Methodist Hospital, Columbus, OH, USA; Pathology, Ohio Health Riverside Methodist Hospital, Columbus, OH, USA

## Abstract

Hemangiomas are rarely found in the axilla, with the most commonly identified axillary mass being lymphadenopathy. We report a unique case report of a post-partum female with an axillary mass that became larger and symptomatic while breastfeeding. On imaging, the mass was found to be complex and cystic, and aspiration was attempted several times. With a rapid return of swelling and worsening symptoms, there was concern for bleeding into the cystic cavity. Ultimately, the >10 cm mass had to be formally excised in the operating room, yielding definitive relief of symptoms. Final pathology reported the mass as a vascular malformation, either a hemangioma or arteriovenous malformation. It has been postulated that estrogen and progesterone may stimulate the growth of hemangiomas, which may explain this patient’s post-partum presentation. This case demonstrates a perplexing axillary mass that continued to re-accumulate until final excision.

## INTRODUCTION

The most commonly reported axillary mass seen on mammography is lymphadenopathy [1–3]. Axillary masses are more frequently benign than malignant [3, 4]. Hemangiomas and arteriovenous malformations have been reported in the breast, however, few case reports exist on such masses in the axilla [5–7]. It has been postulated that estrogens may stimulate the growth of these lesions. Here, we present a case report on the diagnosis and management of a symptomatic axillary cystic mass in a post-partum female, which rapidly reoccurred until operative excision.

## CASE REPORT

**Figure 1:**
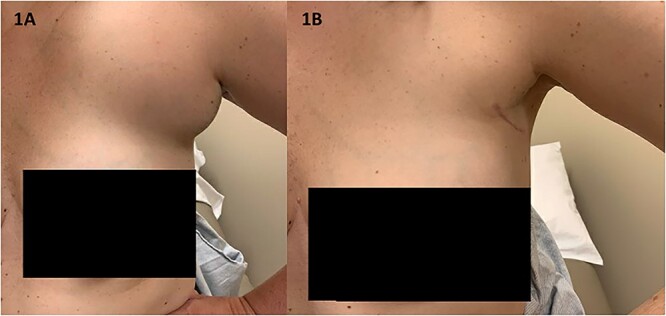
(**A**) The patient’s axilla on initial presentation had significant swelling and tenderness without overlying erythema or other skin changes (**B**) the patient’s axilla at her 2-month post-operative visit demonstrates her well-healed surgical scar and decreased fullness to the area.

**Figure 2:**
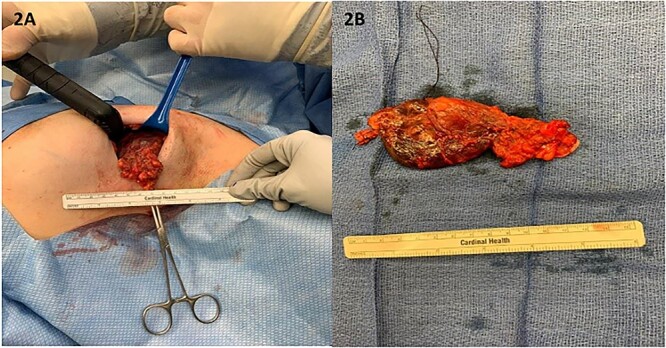
(**A**) Intra-operatively, the mass was lobular in nature and was noted to extend to the axillary vein; (**B**) the mass measured over 10 cm in its longest diameter once fully excised.

**Figure 3:**
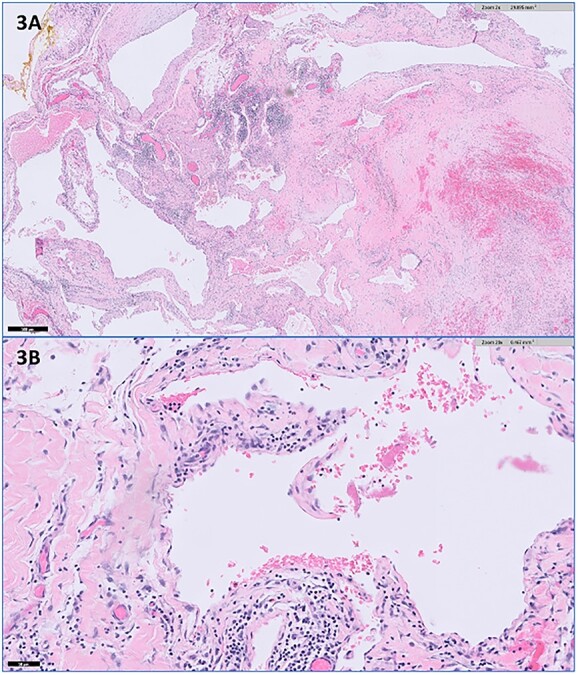
Representative histopathologic images of the vascular malformation with hematoxylin and eosin stain showing ectatic vessels with luminal red blood cells and lined by flattened endothelial cells at (**A**) 2× magnification and (**B**) 20× magnification.

A 33-year-old premenopausal female presented with concern for accessory breast tissue in her left axilla (Fig. 1A). The area was enlarging after her recent pregnancy and with current breastfeeding. It became increasingly tender. She had no prior history of breast disease, breast biopsies or hormone replacement. She was previously on birth control. On exam, the patient had a prominent area of tissue involving her left axilla, measuring 9 × 10 cm. There was no erythema, induration, dimpling or lymphadenopathy. An ultrasound (US) demonstrated a complex cystic mass in the left axilla, measuring 9.7 × 8.2 × 6.5 cm. The cyst was aspirated after core needle biopsy was unsuccessful, yielding 225 ml of serosanguinous fluid with some remaining internal debris. No malignant cells were present on fluid analysis. The patient returned the next day with worsening pain and firmness to her left axilla. There was now concern for bleeding into cyst cavity after aspiration. An additional aspiration was performed with another 140 ml of sanguinous fluid obtained and subsequent cyst collapse. Again, the swelling and pain recurred overnight. A repeat US showed a slightly increased avascular collection (11.6 × 9.5 × 6.7 cm), now with internal echos (previously anechoic and simple), and a central nodular area concerning for clot. A computed tomography of the chest again demonstrated the complex cystic axillary mass (9.9 × 7.7 × 8.7 cm) with a central retracted clot, no active contrast extravasation and the left axillary artery posteriorly. The patient’s pain continued to worsen, now extending into her back and neck. On exam, the swelling had increased into the upper outer quadrant of the left breast. The patient was admitted to the hospital for drainage by interventional radiology. Hemoglobin was unremarkable. A 10 French drain was placed, and 180 ml of dark blood was collected. Cultures were negative. The patient had relief of her symptoms the next day. One week later, the patient underwent excision of left axillary cystic mass and new drain placement. The mass was surrounded by small vessels and extended superior to the left axillary vein. It measured >10 cm once excised (Figs 2A and B). The final pathology identified it as a vascular malformation, likely a hemangioma versus an arteriovenous malformation (Fig. 3). The patient had an uneventful post-operative course with drain removal 10 days later. No further fullness or swelling was reported.

## DISCUSSION

Because the axilla is often visualized on imaging of the breast, numerous types of axillary masses have been reported in the literature. The most frequently identified mass is lymphadenopathy at around 80% [3]. This ranges from benign or reactive lymph nodes to metastatic lymph nodes from a breast or distant primary malignancy [1–3]. There is a paucity of literature on hemangiomas of the axilla [5–7]. Hemangiomas are usually seen in younger women and in the craniofacial region (60%). Approximately, 25% lie on the trunk, and 15% are found on the extremities [3]. Sonographically, hemangiomas appear as well-circumscribed, hypoechoic, lobulated masses [3, 7, 8]. They are usually avascular, although they may have varying degrees of echotextures and vascularity [3, 6–8]. This description correlates well with our US findings, and we did have clot formation on subsequent imaging after aspiration. If we had obtained a mammogram, a hemangioma would appear as a hyperdense or isodense well-circumscribed superficial lesion, sometimes with calcifications [3, 7, 8].

**Figure 4:**
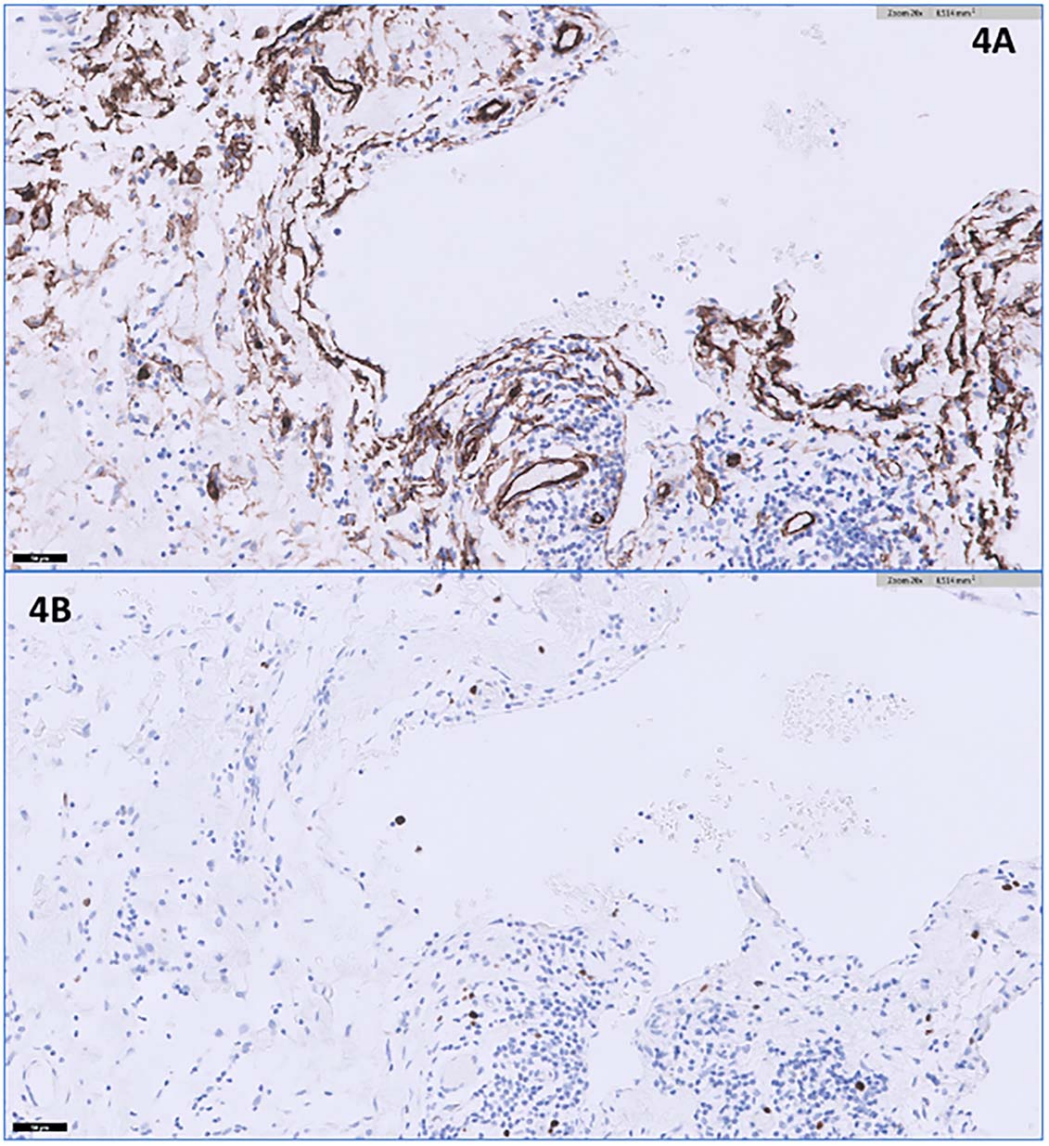
(**A**) The CD34 immunohistochemical stain appears positive lining the endothelial cells (20× magnification); (**B**) the Ki-67 immunohistochemical stain shows a very low proliferation index in lining endothelial cells at <1% (20× magnification).

A core needle biopsy was first attempted; however, it was unsuccessful as the mass was mainly cystic. A fine needle aspiration (FNA) was then performed, which demonstrated lymphocytosis without malignant cells. Utilizing FNA for the biopsy of axillary masses is highly sensitive and specific, particularly in ruling out malignancy [4]. Various markers have been found to be positive in hemangiomas, such as CD34 for vascular endothelium [5–7]. Figure 4A appropriately demonstrates the presence of CD34 staining to a substantial portion of our specimen. The Ki-67 proliferation index is usually low with hemangiomas compared to angiosarcomas [7]. In our specimen, the Ki-67 index was <1% (Fig. 4B), further supporting our diagnosis of hemangioma.

Our patient reported growth of her axillary mass with pregnancy. It was been postulated that estrogen and progesterone exposure can stimulate the growth of various hemangiomas [6, 9–11]. This phenomenon has been seen with both increased sex hormones in pregnancy and after exogenous hormone therapy. The relationship between hormone levels and hemangioma growth is still unclear in the literature, and the presence of increased hormone receptors has been inconsistently proven [10, 11]. What is most remarkable about this case is the rapid re-accumulation of fluid within the cyst, the symptomatic nature of it and the immediate relief of the patient’s symptoms with drainage. The patient experienced a worsening of symptoms and return of swelling nearly overnight following both aspiration procedures. The patient did not have definitive relief until drain placement and then subsequent excision in the operating room. At the patient’s last visit nearly 2 years post-operatively, she was doing well without recurrence of the mass or her symptoms. Figure 1B shows her axilla 2-months post-operatively.

Here, we report the diagnostic work-up and management of a unique axillary cystic mass that was ultimately identified as a hemangioma. Our management of this case was complicated by inconclusive aspiration findings and quickly recurring symptoms. It was not until definitive excision that a hemangioma was identified. There is a wide range of benign and malignant pathology that can be found within the axilla, and the management of such axillary masses can be perplexing as this unique case report demonstrates.
